# Ferritin – a multifaceted protein scaffold for biotherapeutics

**DOI:** 10.1038/s12276-022-00859-0

**Published:** 2022-10-03

**Authors:** Na Kyeong Lee, Seongeon Cho, In-San Kim

**Affiliations:** 1 Chemical and Biological Integrative Research Center, Biomedical Research Institute, Korea Institute of Science and Technology, 5 Hwarang-ro 14-gil, Seongbuk-gu, Seoul, 02792 Republic of Korea; 2 KU-KIST Graduate School of Converging Science and Technology, Korea University, 145 Anam-ro, Seongbuk-gu, Seoul, 02841 Republic of Korea

**Keywords:** Drug development, Therapeutics, Drug development

## Abstract

The ferritin nanocage is an endogenous protein that exists in almost all mammals. Its hollow spherical structure that naturally stores iron ions has been diversely exploited by researchers in biotherapeutics. Ferritin has excellent biosafety profiles, and the nanosized particles exhibit rapid dispersion and controlled/sustained release pharmacokinetics. Moreover, the large surface-to-volume ratio and the disassembly/reassembly behavior of the 24 monomer subunits into a sphere allow diverse modifications by chemical and genetic methods on the surface and inner cage of ferritin. Here, we critically review ferritin and its applications. We (i) introduce the application of ferritin in drug delivery; (ii) present an overview of the use of ferritin in imaging and diagnosis for biomedical purposes; (iii) discuss ferritin-based vaccines; and (iv) review ferritin-based agents currently in clinical trials. Although there are no currently approved drugs based on ferritin, this multifunctional protein scaffold shows immense potential in drug development in diverse categories, and ferritin-based drugs have recently entered phase I clinical trials. This golden shortlist of recent developments will be of immediate benefit and interest to researchers studying ferritin and other protein-based biotherapeutics.

## Ferritin: Delivering more than just iron

Ferritin molecules, hollow protein spheres that store and release iron as needed, also hold promise for disease diagnosis, drug delivery, and vaccine development. Almost all organisms produce ferritin, which consists of 24 protein subunits that can interact with metals. Its biocompatibility, large surface-to-volume ratio, and ease of modification have led to investigation of its use in various medical applications, as reviewed by In-San Kim at the Korea Institute of Science and Technology in Seoul, South Korea, and co-workers. Ferritin can rapidly disperse, and release its contents steadily over a long time. It can deliver diagnostic imaging compounds or drugs to specific cells, or display antigens for immunotherapy or vaccines. Testing is underway for ferritin-based delivery of cancer drugs and development of vaccines for influenza, SARS-CoV-2, and diseases associated with Epstein Barr virus.

## Introduction

The exploitation of protein nanocarriers has played a vital role in advancing disease diagnosis and therapy^1^. Their nanometer size range allows advantageous properties that permit diverse modifications, such as surface functionalization, and enable the rapid dispersion of loaded drugs. Furthermore, protein nanocarriers have improved the targeted delivery and pharmacokinetic profiles of drug cargo. They are also cleared from circulation relatively slowly, which allows controlled/sustained drug release in targeted sites. Due to these advantages, diverse types of protein nanocarriers have been highlighted for their inherent biodegradability and ease of genetic modification compared to other types of nanocarriers^2^. In this review, we highlight ferritin nanocages.

Ferritin exists in almost all living organisms, participating in iron homeostasis by storing and releasing iron ions^3^. Ferritin has excellent biosafety profiles, and the nanosized particles exhibit rapid dispersion and controlled/sustained release pharmacokinetics. Moreover, the large surface-to-volume ratio and the disassembly/reassembly feature of the 24 monomer subunits into a sphere allow diverse modifications on the surface and inner cage of ferritin by chemical and genetic methods. Its hollow spherical structure that naturally stores iron ions has been diversely exploited by researchers in biotherapeutics^4^. Examples of drugs and agents loaded onto ferritin include chemotherapeutic agents, genes, fluorescent molecules, and various peptides that have been displayed on their surface. Optimizing drug loading efficiency is an ongoing task for all delivery vehicles; however, there have been diverse attempts to load various poorly soluble drugs into ferritin. Furthermore, the threefold symmetry axis of the ferritin structure has also allowed antigenic display in its correct conformation, a unique feature of ferritin compared to other particles^5^.

This review summarizes ferritin and its performance in various fields of medicine (Supplementary Fig. 1). The overall aim is to present a short list of the most interesting current and recent approaches to using ferritin in nanomedicine. Here, we critically review ferritin and its applications: (1) the application of ferritin in therapeutics; (2) the application of ferritin in imaging and diagnosis; (3) ferritin-based vaccines in immunotherapy; and (4) current and impending clinical trials of ferritin-based agents. This review of recent developments will be of immediate benefit and interest to researchers studying ferritin and other protein-based biotherapeutics.

## Ferritins in biotherapeutics

The hollow spherical core of ferritin allows the loading of various cargo. This is generally achieved via the mineral pores on the surface or pH-mediated disassembly/reassembly of the nanocage. There have been many investigations, mainly in anticancer therapy that attempted the targeted delivery of chemotherapeutic agents to tumors using ferritin as a delivery vehicle. Most of these studies resulted in successful tumor growth inhibition, superior pharmacokinetic profiles and diminished adverse effects compared to the free drug in both in vitro and in vivo models. Substantial progress has been made in the development of ferritin nanocages for use in drug delivery for cancer therapy.

Doxorubicin-loaded ferritins have shown successful tumor growth inhibition in numerous mouse cancer models by many groups. In one example, Dox-loaded ferritin targeted and was internalized by transferrin receptor 1 (TfR1)-overexpressing tumor cells to mediate 10-fold higher intracellular levels compared to free doxorubicin^6^. Moreover, the encapsulation of paclitaxel in ferritin showed potent apoptosis of MDA-MB-231 breast cancer cells in in vivo models^7^. This is an example of insoluble drug delivery via ferritin, with specific targeting to tumor cells alleviating the adverse effects of this chemotherapeutic agent. Similarly, gemcitabine was loaded onto ferritin and coadministered for photothermal therapy, showing effective adjunctive therapy against breast cancer models^7^. Effective delivery of cisplatin was also achieved by ferritin, resulting in an improved therapeutic index of antiblastic therapy in an advanced, refractory melanoma model^8^. Specific delivery of chemotherapeutic agents to cancer cells by ferritin has resulted in enhanced therapeutic benefit to numerous types of cancer, with some successful in vivo results thus far (Table 1).

**Table 1:** Use of ferritin for chemotherapy.

Encapsulated drug	Target disease	Therapeutic effect	Ferritin	References
Cisplatin	Melanoma	Improved therapeutic index	Human HFn	^8^
Co(II)	Melanoma	Tumor cell apoptosis	Human HFn	^34^
Doxorubicin	Gastric cancer	Tumor growth inhibition (91.1%) (TfR1^+^ cells)	Human HFn	^35^
Doxorubicin	Liver cancer	Reduced cell viability in Hepa1-6 cells after PTT	Apoferritin (Au nanoshell)	^36^
Doxorubicin	Breast cancer	Tumor growth inhibition	Human HFn (CD-modified)	^37^
Doxorubicin and ADNIR	Colon cancer	Tumor growth inhibition (80.8%)	Apoferritin	^38^
Doxorubicin (Cu(II complex)	Glioblastoma	Tumor growth inhibition (89.6%)	Human HFn	^6^
Doxorubicin, RB	Breast cancer	Cell inhibition rate 83%	Apoferritin	^39^
Epirubicin, DBN	Breast cancer	Killed 80% of cancer stem cells (PDT)	Apoferritin	^40^
Gefitinib	Breast cancer	In vitro tumor inhibition	Human HFn	^41^
Gemcitabine (GEM)	Breast cancer	PB-Ft NPs damaged 4T1 cells	Human HFn (PB-modified)	^7^
Paclitaxel	Breast cancer	Tumor growth inhibition (64.6%)	Human HFn	^7^

*TGI* tumor growth inhibition, *TfR1* transferrin receptor-1, *PTT* photothermal therapy, *CD* carbon dot, *ADNIR* ADS-780 near-infrared (NIR) fluorescent dye, *RB* rose bengal, *DBN* 1,1-dioctadecyl-3,3,3,3-tetramethylindotricarbocyanine iodide, *CSCs* cancer stem cells, *PDT* photodynamic therapy, *PB* Prussian blue.

As mentioned above, the ferritin nanocage is composed of 24 monomer subunits, which allows the display of 24 separate peptides if arranged correctly by genetic modification. This has allowed diverse approaches in displaying various immune-stimulating peptides on the ferritin surface for the development of effective immunotherapeutic agents (Table 2). This is a unique advantage of ferritin nanocages and allows the delivery of various (and multiple) peptides via one-step modification^9^. For example, the trimeric peptide display arrangement of the surface of ferritin allows the optimal delivery of TRAIL peptides. More than one successful attempt has been made to deliver this apoptosis-inducing signal on the surface of ferritin nanocages^10–12^.

**Table 2:** Use of ferritin for immunotherapy.

Immunotherapy agent	Target disease	Therapeutic effect	Ferritin	References
PD-L1pep1	All types	Promotion of PD-1 immune checkpoint	Human HFn	^42^
PD-1	All types	Promotion of T-cell activation in the lymph node	Human HFn	^43^
SIRPα	Colon cancer	Effective tumor growth inhibition, tumor-specific CD8^+^ T-cell activation	Human HFn	^13^
Trimer-mimetic TNF superfamily ligand	All types	Effective induction of apoptosis of tumor cells in in vivo model	Human HFn	^10^
Trimeric TRAIL	All types	Tumor growth inhibition in breast and pancreatic cancer model	Human HFn	^11^
Tumor-specific antigens or IC molecules	All types	TSA-specific CD8^+^ T-cell activation	Human HFn	^11^

*PD-L1pep1* PD-L1 binding peptide, *PD-1* programmed-cell death receptor 1, *SIRPα* signal-regulatory protein alpha, *TNF* tumor necrosis factor, *TRAIL* TNF-related apoptosis-inducing ligand, *IC* immune checkpoint molecule, *TSA* *tumor-specific antigens*.

In another interesting work, the phagocytosis-inducing peptide SIRPα was displayed by doxorubicin-loaded ferritin^13^ to achieve an intrinsic vaccination effect. By the cross priming of effector CD8^+^ T cells, the simultaneous delivery of SIRPα and doxorubicin, an immunogenic cell death (ICD)-inducer, achieved potent tumor growth inhibition in a melanoma model and even against tumor rechallenge in a colon cancer model. This study attempted to trigger the presentation of cancer cell neoantigens to the host immune system, facilitating the persistent amplification of antitumour T cells, which resulted in an especially interesting and effective therapeutic approach. There are also other interesting approaches using ferritin in addition to the abovementioned studies, such as a study by Seo et al. that proposed a thrombolytic ferritin expressing multivalent clot-targeting peptides and fibrin degradation enzymes for coadministration with chemotherapy^14,15^. Similarly, a study by Lee et al. used γ-carboxyglutamic acid of protein C (PC-Gla) and thrombin receptor agonist peptide (TRAP) to treat acute inflammatory sepsis in in vivo mouse models^16^.

## Ferritins in imaging and diagnosis

Ferritin nanocages have been readily modified to develop diagnostic agents for various imaging methods (computer tomography, CT/magnetic resonance imaging MRI). Fluorescent molecules can be incorporated or loaded as cargo at the same time as targeting peptides on the ferritin surface for targeting disease biomarkers^17^. This would allow multimodal imaging techniques for ferritin-based agents with enhanced diagnostic accuracy, and the ferritin-based agents developed are listed below (Table 3).

**Table 3:** Use of ferritins in imaging and diagnosis.

Application	Ferritin	Cargo	Modification	References
Fluorescence imaging	Short ferritin	Tumor-targeting proapoptotic peptide, GFP	Genetic modification	^44^
CT imaging,	Horse spleen ferritin	Bi_2_S_3_	Incubation (Inlaying)	^7^
Peroxidase nanoenzyme	*Pyrococcus furiosus* ferritin	Co_3_O_4_	Mineralization	^45^
Fluorescence imaging, MRI	Human HFn	Cy5.5, Fe_3_O_4_	Chemical conjugation, mineralization	^24^
SPECT, MRI	Human HFn	Fe_3_O_4_, 125I	Mineralization, chemical conjugation (Iodogen method)	^18^
Peroxidase nanoenzyme	Horse spleen ferritin	Prussian blue	Mineralization	^46^
MRI	Horse spleen ferritin	Mn(III)OOH	Mineralization	^47^
Fluorescence imaging, MRI	*Archaeoglobus fulgidus* ferritin	GQDs, Fe	Disassembly/reassembly	^48^
Fluorescence imaging, MRI	Human HFn	GFP, Fe_3_O_4_	Genetic modification, mineralization	^49^
Fluorescence imaging	Human HFn	RFP	Genetic modification	^21^
Fluorescence imaging	Human HFn	Indocyanine Green	Disassembly/reassembly	^23^
Fluorescence imaging, PAI	Human HFn	Tricarbocyanine	Disassembly/reassembly	^19^
Fluorescence imaging	Human HFn	ZnF16Pc, ZW800	Disassembly/reassembly, chemical conjugation	^6^
PET imaging, fluorescence imaging	Human HFn	Cy5.5, 64Cu	Chemical conjugation, genetic modification, disassembly/reassembly	^50^
PET imaging, MRI, PAI	Horse spleen ferritin	Melanin, 64Cu	Disassembly/reassembly, incubation	^51^
Fluorescence imaging	Human HFn	Cy5.5, BHQ-3	Bioconjugation, chemical conjugation, disassembly/reassembly	^50^

*CT* computed tomography, *MRI* magnetic resonance imaging, *SPECT* single-photon emission computerized tomography, *GQD* graphene quantum dot, *GFP* green fluorescent protein, *RFP* red fluorescent protein, *PAI* photoacoustic imaging, *ZW800* zwitterionic near-infrared fluorophore, *PET* positron emission tomography, *BHQ* black hole quencher.

For example, magnetoferritin probes consisting of iron oxide and 125I radionuclides on the ferritin surface were developed for the multimodal imaging of tumors by Zhao et al.^18^. Moreover, a study by Huang et al. developed near-infrared dye IR820-loaded ferritin for fluorescence and photoacoustic photothermal therapy with high imaging contrast^19^. Another study by Crich et al. developed gadolinium-loaded ferritin for contrast MRI, which allowed the imaging of tumoral endothelial cells^20^.

More common fluorescent molecules, such as red fluorescent protein (RFP), have been loaded in a study by Lee et al., where tumor-specific antigens were simultaneously delivered to the lymph nodes in mouse models^21^. Another example is a study by Lin et al. that developed hybrid ferritin probes for tumor cell-targeted near-infrared fluorescence (NIRF) imaging^22^. Similarly, indocyanine green (ICG)-loaded ferritin was developed by Sitia et al. for tumor-specific imaging that showed clinical relevance for fluorescence imaging-guided surgery of cancer^23^. In addition, fluorescent Cy5.5 was loaded onto magnetic ferritin, which targeted vascular macrophages in imaging in vivo inflammation^24^. As shown by these examples, ferritin is a multifunctional platform that can also target specific cells/biomarkers.

## Ferritin-based vaccines in immunotherapy

Ferritin-based vaccines have attracted considerable interest due to their potency and safety^25^. Conventional vaccines composed of inactivated viruses or organisms carry the potential risk of triggering reversion, and thus, attempts to develop more immunogenic yet safe vaccines continue^26^. Antigen display on the ferritin surface has many desirable features, such as the uniform presentation of 24 epitopes, as well as monodispersity and thermal and pH stability of the ferritin nanocage. Furthermore, the particle-mediated delivery of peptides has been shown to trigger more potent stimulation than soluble peptides^27^. Ferritin nanocages, due to their size (10~12 nm), can be readily taken up by dendritic cells (DCs) for migration to the lymph node to augment cellular and humoral immune responses. Due to these numerous advantages, ferritin-based vaccines have proven especially potent and can be applied not only to infectious diseases but also to cancer vaccines and vaccines for autoimmune diseases.

Representative ferritin-based vaccines target influenza, SARS-CoV-2 and Epstein‒Barr viruses, and some have entered phase I clinical trials^26,28^. Ferritin-based vaccines have proven biocompatible yet immunogenic with no significant adverse effects. However, the challenging features of ferritin-based vaccine development are nanoparticle heterogeneity, inadequate folding of antigens and intersubunit interactions resulting in antigen interference. Since antigens are encoded onto the ferritin protein scaffold, the self-assembled expression and purification of ferritin-based vaccines still require careful optimization. The selected clinical trials of ferritin nanocages currently in vaccine development are listed below (Table 4).

**Table 4:** Ferritin-based vaccines.

Application	Ferritin	Antigen	Target	References
DC-targeting	*P. furiosus*	OT-1, OT-2	T-cell receptors OT-1, OT-2	^19^
Tumor targeting	Human HFn	RGD4C peptide	Tumor vasculature (α_v_β_3_ integrin)	^52^
Tumor targeting	Human HFn	RFP	RFP-expressing melanoma	^21^
Tumor targeting	Human Fn	HPV16 E7 peptide	MC38 colon cancer	^7^
Viral vaccination	*H. pylori*	S protein	SARS-CoV-2	^28^
Viral vaccination	*H. pylori*	S protein	SARS-CoV-2	^32,53^
Viral vaccination	*H. pylori*-bullfrog hybrid	RBD of S protein	SARS-CoV-2	^15^
Viral vaccination	−	S protein	SARS-CoV-2	^23^
Viral vaccination	*E. coli* K12	hRID-RBD of S protein	MERS-CoV	^54^
Viral vaccination	*H. pylori*-bullfrog hybrid	RBD of HA	Influenza virus	^55^
Viral vaccination	*H. pylori*-bullfrog hybrid	gp350(D_123_)	EBV	^33^
Viral infection	*H. pylori*	VP6	Rotavirus A	^7^
Bacterial infection	*H. pylori*-bullfrog hybrid	OspA	*B. burgdorferi*	^56^

OT-1/-2 CD8^+^ and CD4^+^ T-cell epitopes corresponding to res 257-264 and 323-339 of ovalbumin, respectively, *RGD4C* active peptide targeting the αvβ3 integrins, *RFP* red fluorescent protein, *HPV16* human papillomavirus type 16, *MC38* murine colon adenocarcinoma, *S protein* spike protein from severe acute respiratory syndrome coronavirus 2 (SARS-CoV-2), *MERS-CoV* Middle East respiratory syndrome coronavirus, *RBD* receptor binding domain, *HA* hemagglutinin from influenza virus, *gp350(D*_*123*_*)* res 2–415 of the ectodomain of glycoprotein 350/220 from Epstein‒Barr virus (EBV), *VP6* intermediate capsid protein of human rotavirus A, *OspA* lipoprotein on *Borrelia burgdorferi* outer membrane surface when the bacgteria reside in the tick gut.

## Ferritins in clinical trials

Clinical trials with ferritin-based vaccines are in the early stages (Table 5). One example is the work of Kanekiyo et al.^29^, in which trimeric hemagglutinin (HA) was fused to the 3-fold axis of ferritin, giving the display of eight trimeric viral spikes. The ferritin-HA showed a potent immunological effect (10-fold higher antibody titers) compared to an inactivated vaccine. The promising results obtained with this design have motivated the development of three vaccines against influenza to be tested in Phase 1 clinical trials (ClinicalTrials.gov ID NCT03186781, NCT03814720, and NCT04579250)^29,30^.

**Table 5:** Ferritin in clinical trials.

Agent	Disease	Results	Phase/Status	Trial number	References
Ferritin-HA	Influenza	Seroconversion rates of 40% or 90% and IC50 titers of 1 × 10^3^ or 3 × 10^3^	I/Completed	NCT03186781	^31^
Ferritin-HA	Influenza	–	I/Completed	NCT03814720	^29,30^
Ferritin-HA	Influenza	–	I/Ongoing	NCT04579250	^29,30^
Ferritin-gp140	SARS-CoV-2	–	I/Ongoing	NCT04784767	^32^
Ferritin-gp350	EBV	–	I/Recruiting	NCT04645147	^33^

Positive results have been obtained from the first trial for influenza, NCT03186781, where the seroconversion rates were 40% or 90%, and the 50% inhibitory concentration (IC50) titers were 1 × 10^3^ and 3 × 10^3^ for monotherapy and coadministration with an influenza DNA vaccine, respectively^31^. No significant side effects were reported. The ferritin-HA vaccine was followed by the development of other ferritin-based vaccines targeting severe acute respiratory syndrome coronavirus 2 (SARS-CoV-2). Although no results have been posted yet, a single administration of this vaccine was effective in mouse models, and this study has also entered phase I clinical trials (ClinicalTrials.gov ID NCT04784767)^32^.

The third ferritin-based vaccine targets Epstein‒Barr virus (EBV) and has also entered a phase 1 clinical trial (ClinicalTrials.gov ID NCT04645147). In this vaccine, the surface of ferritin is decorated with gp350 (glycoprotein 350/220 of EBV), and the neutralization capacity was reported to be significantly higher than that of soluble gp350 in mouse models^33^.

## Conclusion

Protein nanocarriers contribute numerous advantages to the field of disease diagnosis and drug development, and current approaches in diverse categories of biotherapeutics, immunotherapy, and vaccines will in no doubt provide therapeutic benefit. Ferritin has excellent biocompatibility and biodegradability and provides many possibilities for modification. The subunit structure of ferritin allows the uniform display of 24 peptides on its surface, which can be achieved by one-step genetic modification or direct conjugation via chemical methods. Furthermore, the triggering of a more potent response by the particle-mediated delivery of peptides is a well-known phenomenon in peptide delivery, as is the triggering of immunomodulatory responses. Additionally, the hollow cage structure allows the delivery of various poorly soluble or cytotoxic drugs by disassembly/reassembly or mineralization through surface pores. Ferritin is an all-in-one multifunctional protein scaffold.

The major strength of ferritin nanocages in nanomedicine is in vaccine development. Three vaccines against influenza have entered phase I clinical trials, and one has provided positive results. The efficacy of ferritin-based vaccines has triggered investigations into optimizations for developing other ferritin-based vaccines in clinical trials. Ferritin holds great potential in disease diagnosis, prevention and therapy.

## Supplementary information

Supplemental Material File #1

## Data Availability

All data are included in this published article.
