# Supplementary material for: Ferritin – a multifaceted protein scaffold for biotherapeutics
Source: Exp Mol Med. 2022 Oct 3;54(10):1652–7. doi: 10.1038/s12276-022-00859-0 (PMC9527718; doi:10.1038/s12276-022-00859-0)
Supplement: Supplementary file 1 — Supplemental Material File #1 [file 12276_2022_859_MOESM1_ESM.pdf]

Experimental & Molecular Medicine

Ferritin – a multifaceted protein scaffold for biotherapeutics

Author Information

Affiliations

Author 1 Na Kyeong Lee, Ph.D.

Author 2 Seongeon Cho

Author 3 In-San Kim, M.D., Ph.D.

Center for Theragnosis, Biomedical Research Institute, Korea Institute of Science and Technology, 5 Hwarang-ro 14-gil, Seongbuk-gu, Seoul, 02792, Republic of Korea

Author 1, Author 2 & Author 3

KU-KIST Graduate School of Converging Science and Technology, Korea University, 145 Anam-ro, Seongbuk-gu, Seoul, 02841, Republic of Korea

Author 2 & Author 3

Contributions

N.K.L., conceptualization and writing; S.C., conceptualization and writing; and I-S.K., writing – reviewing and editing and funding acquisition.

Corresponding author

Correspondence to: Author 3

Supplementary Information

Supplementary Fig. 1

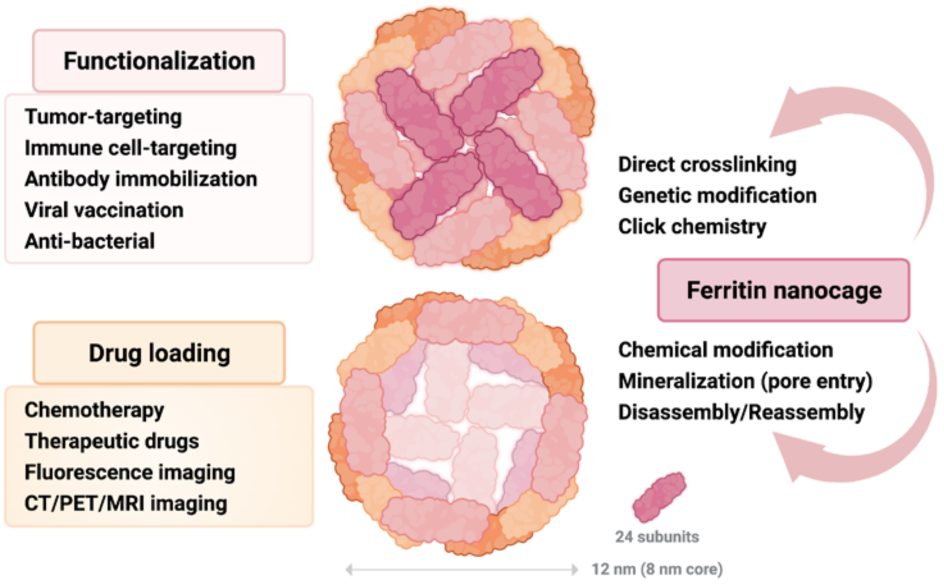

Functionalization and drug loading strategies for ferritin nanocage. Ferritins allow easy genetic and chemical modification either on its surface and the inner hollow cavity for various purposes in research and biomedicine (created with Biorender.com).
